# Supplementary material for: Lercanidipine Synergistically Enhances Bortezomib Cytotoxicity in Cancer Cells via Enhanced Endoplasmic Reticulum Stress and Mitochondrial Ca2+ Overload
Source: Int J Mol Sci. 2019 Dec 4;20(24):6112. doi: 10.3390/ijms20246112 (PMC6941136; doi:10.3390/ijms20246112)
Supplement: Supplementary file 1 [file ijms-20-06112-s001.pdf]

Supplementary Information

Supplementary Figures

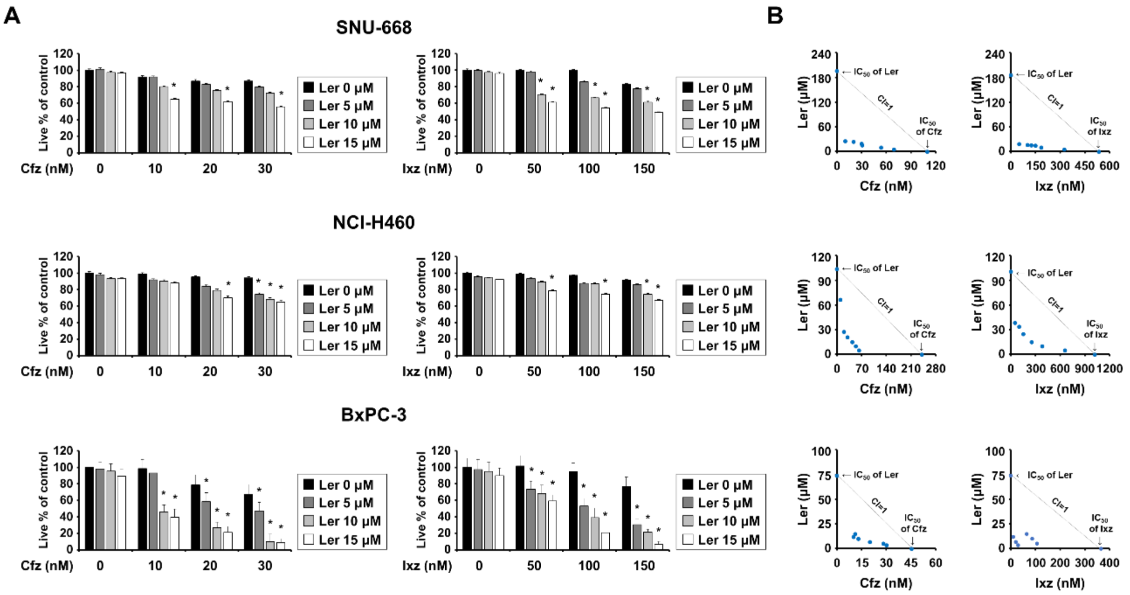

Figure S1. Ler sensitizes various cancer cells to PIs-mediated cell death (**A**) Cells were treated with the indicated concentrations of PIs and/or Ler for 24 h and cellular viability was assessed using the IncuCyte as described in Materials and Methods. The percentage of live cells was normalized to that of untreated control cells (100%). Data represent the means ± S.D. ($n = 7$). One-way ANOVA and Bonferroni's post hoc test. * $p < 0.001$ vs PI treated cells. (**B**) Isoboles for the combination of PIs and Ler that proved iso-effective (IC_{50}) for inhibiting cell viability.

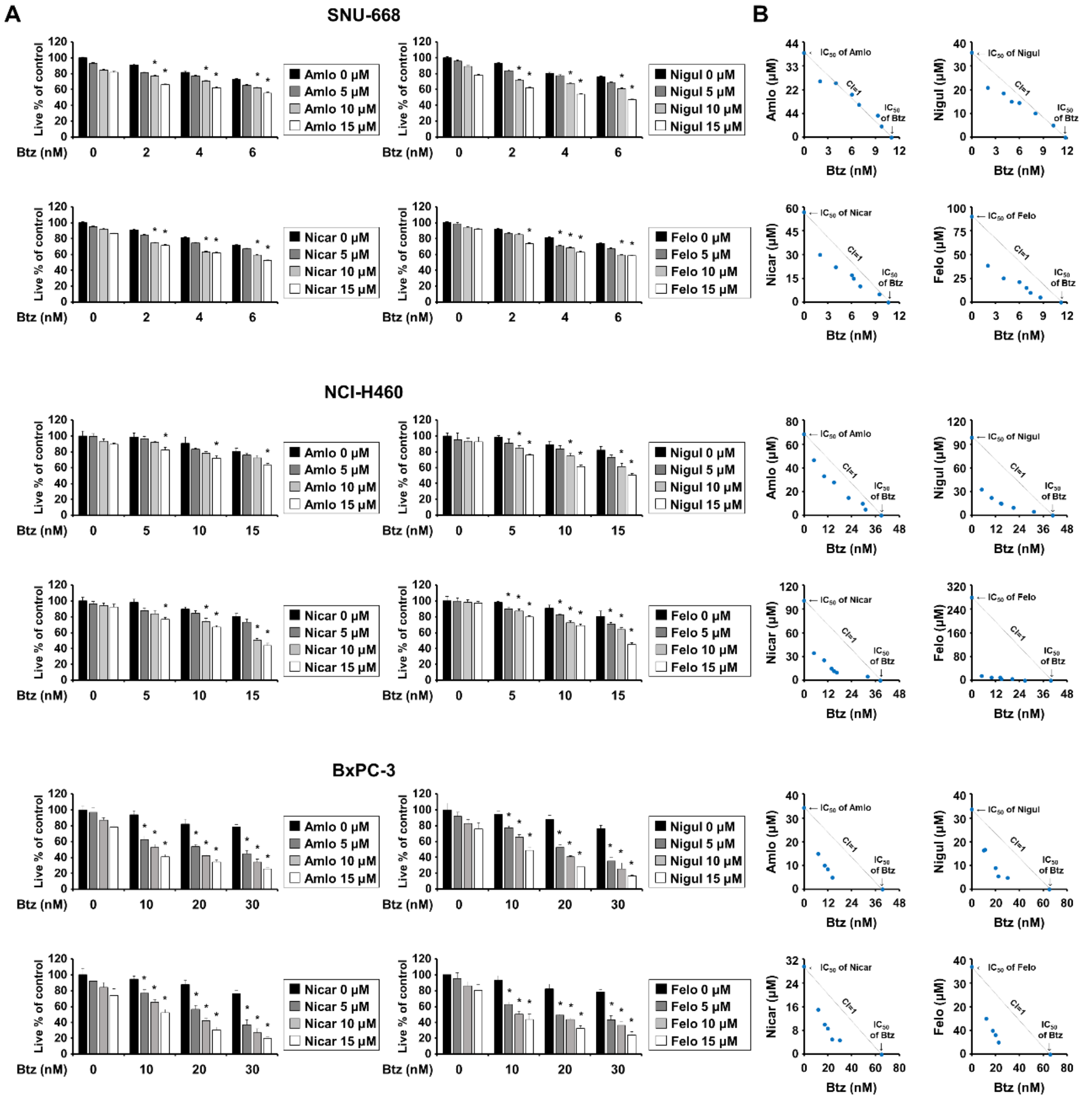

Figure S2. Combinations of each DHP and Btz selectively induce cell death in various cancer cells. (**A**) Cells were treated with the indicated concentrations of Btz and/or DHPs for 24 h and cellular viability was assessed using the IncuCyte as described in Materials and Methods. The percentage of live cells was normalized to that of untreated control cells (100%). Data represent the means ± S.D. (n = 7). One-way ANOVA and Bonferroni's post hoc test. * $p < 0.001$ vs PI treated cells. (**B**) Isoboles for the combination of PIs and Ler that proved iso-effective (IC_{50}) for inhibiting cell viability.

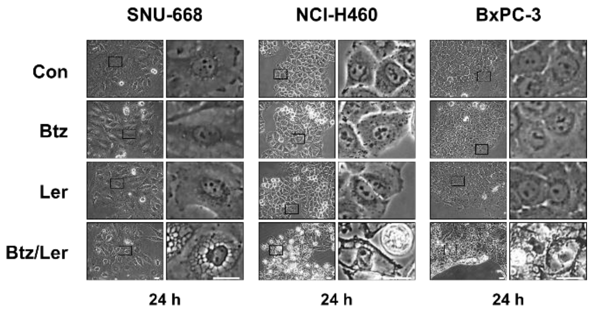

Figure S3. Combination of Btz and Ler induces cytoplasmic vacuolation in various cancer cells. Cellular morphologies were observed by phase-contrast microscopy. Bars, 20 μm. Cells were treated with Btz and/or Ler (for SNU-668 cells, 4 nM Btz and/or 10 μM; for NCI-H460 cells, 15 nM Btz and/or Ler 10 μM; for BxPC-3 cells, 20 nM and/or 10 μM Ler) for 24 h. Bars, 20 μm.

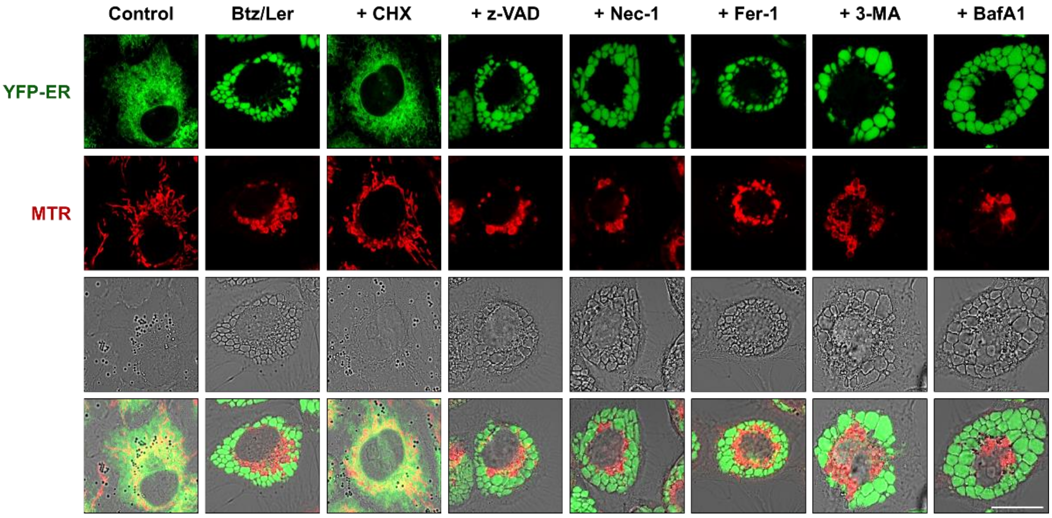

Figure S4. Btz/Ler-induced dilation of mitochondria and the ER does not depend on apoptosis, necroptosis, ferroptosis, or autophagy. YFP-ER cells were untreated or pretreated with 2 μM CHX, 20 μM z-VAD, 20 μM Nec-1, 1 μM Fer-1, 0.25 mM 3-MA, or 10 nM BafA1 and further treated with 4 nM Btz and/or 10 μM Ler for 12 h. Cells were stained with MTR and observed by confocal microscopy. Bars, 20 μm.
